# Adherent-invasive *Escherichia coli* (AIEC) in pediatric Crohn’s disease patients: phenotypic and genetic pathogenic features

**DOI:** 10.1186/1756-0500-7-748

**Published:** 2014-10-22

**Authors:** Maria Pia Conte, Catia Longhi, Massimiliano Marazzato, Antonietta Lucia Conte, Marta Aleandri, Maria Stefania Lepanto, Carlo Zagaglia, Mauro Nicoletti, Marina Aloi, Valentina Totino, Anna Teresa Palamara, Serena Schippa

**Affiliations:** Department of Public Health and Infectious Diseases, ‘Sapienza’ University of Rome, Rome, Italy; Departmen of Experimental and Clinical Sciences, University “G. D’Annunzio” Chieti, Chieti, Italy; Department of Pediatrics, University of Rome “La Sapienza”, Rome, Italy; Department of Public Health and Infectious Diseases, Pasteur Institute Cenci-Bolognetti Foundation, Sapienza University, Rome, 00185 Italy; IRCCS San Raffaele Pisana, Rome, 00166 Italy

**Keywords:** Gut microbiota, Pathobionts, Crohn’s disease, Adherent-invasive *E. coli*, AIEC

## Abstract

**Background:**

Adherent-invasive *Escherichia coli* (AIEC) have been implicated in the ethiopathogenesis of Crohn’s disease (CD). In this study, we analyzed a collection of intestinal mucosa-associated *E. coli* isolates, presenting AIEC phenotypes, isolated from biopsies of CD pediatric patients and non-inflammatory bowel diseases (IBD) controls, in order to investigate their genetic and phenotypic pathogenic features.

**Results:**

A total of 616 *E. coli* isolates from biopsies of four pediatric CD patients and of four non-IBD controls were collected and individually analyzed. For AIEC identification, adherent isolates were assayed for invasiveness, and the capacity of the adhesive-invasive isolates to survive and replicate intracellularly was determined over macrophages J774. In this way we identified 36 AIEC-like isolates. Interestingly, their relative abundance was significantly higher in CD patients (10%; 31/308) than in non-IBD controls (1%; 5/308) (*χ*2 = 38.96 p < 0.001). Furthermore pulsed field gel electrophoresis (PFGE) and randomly amplified polymorphic DNA (RAPD) techniques were applied to analyze the clonality of the 36 AIEC-like isolates. The results obtained allowed us to identify 27 distinct genotypes (22 from CD patients and 5 from non-IBD controls). As for the AIEC prototype strain LF82, all 27 AIEC genotypes presented an aggregative pattern of adherence (AA) that was inhibited by D-mannose, indicating that adhesiveness of AIEC is likely mediated by type 1 pili. PCR analisys was used to investigate presence of virulence genes. The results indicated that among the 27 AIEC isolates, the incidence of genes encoding virulence factors K1 (*χ*2 = 6.167 *P* = 0.013), *kps*MT II (*χ*2 = 6.167 *P* = 0.013), *fyuA* (*χ*2 = 6.167 *P* = 0.013), and *ibeA* (*χ*2 = 8.867 *P* = 0.003) was significantly higher among AIEC strains isolated from CD patients than non-IBD controls.

**Conclusions:**

The identification of AIEC strains in both CD and non-IBD controls, confirmed the “pathobiont” nature of AIEC strains. The finding that AIEC-like isolates were more abundant in CD patients, indicates that a close association of these strains with CD may also exists in pediatric patients.

## Background

Crohn’s disease (CD) is a disorder of the human bowel, resulting from chronic inflammation of the gastrointestinal tract. Characteristics of CD are patchy inflammation, full-thickness lesions of the intestinal wall, and granulomas [1, 2]. The etiology of this disease has not been fully elucidated yet although it is generally accepted that the chronic inflammation is due to deregulated immune responses towards the intestinal microbiota [3, 4]. The intestinal microbiota is a dynamic ecosystem that has developed a mutualistic relationship with its host and plays a crucial role in the development and homeostasis of the immune system [5]. Recent studies have suggested that the intestinal microbiota composition contribute to CD pathogenesis since, compared to normal individuals, the microbiota of CD patients display reduced diversity and altered composition [6]. Unbalanced composition of the intestinal microbiota has been defined dysbiosis. A dysbiosis, localized or generalized, has been reported in CD patients and it has been hypothesized that the loss or reduction in the number of beneficial microbes may favor colonization by potentially aggressive pathogenic microorganisms [7, 8]. Whether dysbiosis is a consequence, a cause or a factor contributing to the insurgence, development of the disease remains poorly understood. To date, *Mycobacterium paratuberculosis* and *Escherichia coli* strains have been proposed to be associated with the onset of CD [9]. In particular, increased number of mucosa associated *E. coli* are observed in CD patients [10] and some genotypes, more than others, appear to be associated with the disease [11]. Among these, mucosa-associated *E. coli* strains*,* have acquired a particular relevance to their strong adhesive-invasive properties, [12, 13]. This phenotype, called *E. coli* AIEC (adherent/invasive *E. coli*), has been proposed as a separate pathogenetic category. Several studies conducted mainly with strains isolated from adult CD patients, have suggested that AIEC strains may be involved in the onset and/or in the persistence of CD [14–16]. A detailed study of the prototype of these strains, AIEC LF82 strain, has revealed that it is able to adhere and to invade enterocytes, and to survive and replicate within macrophages without causing host cell death [17]. The adhesion-invasion phenotype of LF82 strain requires the expression of type 1 pili, and of several outer membrane proteins (OMPs), [18–20]. Furthermore, it has been shown that LF82 was able to translocate the intestinal mucosa, thus altering its permeability [21] and to move into deeper tissues activating an inflammatory immune response [22]. LF82 was also shown to be able to escape autophagy both in intestinal epithelial and dendritic cells and to interact with the M cells of Peyer’s patches, through the expression of long polar fimbriae [23, 24]. In silico analysis of the genome of LF82 strain indicates the presence of patho-adaptive mutations whose role in the pathogenicity of this strains have not been fully characterized yet [25]. Furthermore, it has been recently reported that transient colonization of TLR5-deficient mice with AIEC strains, is sufficient to induce intestinal inflammation and dysbiosis [26], reinforcing the hypothesis that AIEC strains likely play a central role in triggering chronic intestinal inflammation in susceptible host. Therefore, to precisely define the role of these bacteria in inflammatory bowel diseases (IBD) we need to deepen our knowledge regarding the virulence traits of these strains, especially for those isolated from pediatric CD patients whose available information is still very limited. In this work we collected and characterized 616 mucosa associated *E. coli* isolates present in ileum biopsies from four CD and four non-IBD pediatric patients (77 *E. coli* isolates per ileal biopsy). The relative abundance of isolates presenting AIEC-like properties was determined. Moreover, genotypic richness and the presence of selected virulence genes of AIEC-like strains were characterized using different molecular approaches.

## Methods

### Patients and bacterial strains

Six hundred-sixteen mucosa-associated independent *E. coli* isolates were collected from ileum biopsies of four pediatric patients with active CD and from four pediatric patients with functional intestinal disorders but presenting normal colonoscopy and histology (non-IBD controls) (age range 2–14 years). Patients were recruited at the Pediatric Gastroenterology Unit of the University “Sapienza” of Rome, Italy. The study protocol was approved by the Committee on Ethical Practice of the ‘Policlinico Umberto I’ hospital. Children were enrolled in the study after written informed consent from their parents. The diagnostic investigation was carried out according to widely agreed international protocols. Infectious and systemic diseases as well as structural abnormalities of the gastrointestinal tract were excluded in all patients. No patient had food allergy or malabsorption,and invasive organisms, parasites and ova were not found in the stools. *Clostridium difficile* or its toxins were not detected in the stool of patients included in the study. No patients had previous treatment with azathioprine/6-mercaptopurine, ciclosporin or other immunosuppressive agents at any time before the enrolment. The four children with CD showed an ileocolonic involvement, and had a disease activity score ranging to moderate to severe. All patients did not receive any antibiotic and corticosteroid treatments within 3 months and four weeks, respectively, before biopsies were taken. Isolation and characterization of independent *E. coli* isolates from biopsy tissues have been described previously [27]. Briefly, each biopsy sample was washed in 500 μl of buffered physiological saline supplemented with 0.016% dithioerythritol to remove the mucus and then shacked four times for 30 s in fresh physiological saline. At this point the biopsy specimens were hypotonically lysed by wortexing for 30 min in 500 μl of distilled water and 100 μl of each lysed suspension was diluted and directly plated onto Mac Conkey plates to obtain well-isolated colonies. Biochemical identification of *E. coli* isolates was carried out with the API 20E system (bio-Merieux-Italia, Rome, Italy) and/or using the indole assay. Seventy-seven *E. coli* isolates per biopsy were subjected to further analysis *E. coli* K-12 reference strain DH5α, *E. coli* EPEC 32 (O55), EIEC strain HN280 [28] and reference *E. coli* AIEC LF82 strain (a kind gift of Arlette Darfeuille-Michaud, Université d’Auvergne, France) were used as positive or negative controls in adhesion-invasion assays. AIEC strain LF82 and *E. coli* reference strains EAEC 042, ETEC EDL 1493, DAEC C1845 and ExPEC CFT073 (provided by Prof. P. Escobar-Paramo; INSERM, Paris, France) were used to compare genotypic and phenotypic characteristics of clinical isolates. Strains were usually grown in Brain Heart Infusion (BHI, Oxoid, Rome, Italy) or on Trypticase Soy Agar plates (TSA, Oxoid) overnight at 37°C. Cultures were stocked at -70°C in the presence of 15% glycerol.

### Cells

Human Caco-2 (ATCC HTB-37) and HEp-2 (ATCC CCL-23) cell lines were used to determine bacterial adhesiveness and invasiveness of the *E. coli* isolates. HEp-2 cells were maintained in Eagle’s minimal essential medium (E-MEM, Sigma, Italy) with 5% heat-inactivated foetal calf serum (FCS, Euroclone Italy) supplemented with 1% penicillin/streptomycin, in 5% CO_2_ atmosphere at 37°C. Caco-2 cells were grown in Minimum Essential Medium (MEM, Euroclone, Milan, Italy), supplemented with 1% penicillin/streptomycin and 10% FCS and maintained in 5% CO_2_ atmosphere at 37°C. The macrophage-like J774A.1 cell line (ATCC TIB-67) was used to determine survival and multiplication in macrophages. J774 macrophages were maintained in RPMI 1640 medium (Euroclone, Italy) supplemented with 10% FCS and 1% penicillin/streptomycin and 5% CO_2_ at 37°C.

### Adhesiveness

All 616 *E. coli* isolates were individually tested for their ability to adhere to cultured cells. Briefly, HEp-2 cell monolayers were cultured on glass coverslips in 24-well plates at a density of 1 × 10^5^ cells/well for 24 h at 37°C. When needed the medium was replaced 3 h before infection with complete medium containing 0.5% (wt/vol) D-mannose (Sigma-Aldrich, Italy). Bacterial cultures were harvested in the exponential phase and suspended in culture medium with and without 0.5% D-mannose. Cell monolayers were infected with bacterial suspensions (10^6^ bacteria/ml; MOI = 10) and incubated at 37°C for 3 h. At the end of incubation period infected monolayers were extensively washed with PBS, fixed in methanol, Giemsa stained and examined with a light microscope. The ratio between the number of adherent bacteria and the number of plated cells was determined counting manually adherent bacteria present in 10 to 20 randomly chosen microscopic fields. A strain was considered to be adherent if it was observed to adhere to more than 40% of cells. *E. coli* AIEC LF82 and *E. coli* K-12 DH5α strains were utilized as positive and negative controls, respectively. Adhesion assays were performed in three independent experiments, in duplicate.

### Invasiveness

The invasive ability of isolates was carried out only with strains presenting an adhesive phenotype. Invasiveness was determined using the gentamicin protection assay, as described by Darfeuille–Michaud et al. [13]. Adhesive strains were then used to individually infect monolayers of HEp-2 cells cultured in 24-well plates at a density of 2 × 10^5^ cells/well. Bacteria were grown overnight in BHI broth at 37°C, and HEp-2 cell monolayers were infected with 0.5 ml of bacterial suspensions (2 × 10^6^; MOI = 10) and incubated for 3 h at 37°C. At the end of incubation period, cell monolayers were washed four times with PBS and fresh medium with 100 μg/ml of gentamicin (Sigma) was added to each well and incubation was continued for 1 h to allow intra-cellular multiplication. Monolayers were extensively washed and lysed with a solution of 1% vol/vol Triton X-100. Dilutions of cell lysates were plated in TSA agar plates to determine the number of viable bacteria (invasive). An isolate was considered invasive when the ratio of the number of intracellular bacteria/initial inoculum X 100 was ≥0.1% (i.e. ≥2 × 10^3^ bacteria/well).

### Survival and replication in J774 macrophages

The macrophages cell line J774 was used as model to assess survival and intracellular multiplication of the adherent/invasive *E. coli* isolates as described by Bringer et al. [29] with minor modifications. Briefly macrophages were seeded in 24-well tissue culture plates at a density of 2 × 10^5^ cells/well, grown for 24 hours at 37°C and infected with adherent/invasive *E. coli* isolates (MOI = 10). After 20 min of incubation at 37°C, infected monolayers were extensively washed with PBS, and supplemented with fresh culture medium containing 100 μg/ml of gentamicin for 40 min at 37°C (1 h post infection). One hour post infection, fresh medium containing 50 μg/ml of gentamicin was added and infected monolayers were further incubated for additional 24 hours. Survival and replication of bacteria within macrophages was determined 1 and 24 h post-infection as described above. Isolates were considered able to survive and multiply in macrophages when the ratio between the number of intracellular bacteria recovered after 24 h of incubation and those recovered after 1 h (X 100) was ≥100%. Bacteria able to replicate within macrophages where those that presented a ratio ≥200%. Adherent/invasive bacteria able to survive and to multiply within macrophage were considered AIEC strains. All assays were performed in three independent experiments, in duplicate.

### PFGE with XbaI-digested genomic DNA

The genetic relationship among AIEC isolates was examined by pulse-field electrophoresis (PFGE). Briefly, bacterial cultures were suspended in TN-buffer (Tris-HCl 50 mM pH 7.4, NaCl 150 mM) and embedded in 1% agarose (LM-MP agarose, Roche, Italy) plugs. Embedded bacteria were lysed by immerging the plugs in a solution of Tris-HCl 200 mM pH 7.4, EDTA 0.5 M pH 8, SDS 10% proteinase K (20 mg/ml, Roche) and incubated for 24 h at 56°C. Agarose plugs were washed once with TE-buffer (Tris-HCl 200 mM pH 7.4, EDTA 0.5 M pH 8) and DNA was digested with 40 U/plug of *Xba*I overnight at 37°C. The restriction fragments were separated by PFGE on a CHEF-DR II system (Bio-Rad Laboratories, Richmond, CA, USA). The pulse time ranged from 1 to 20 seconds over 21 hours (6 V/cm) at 14°C. Lambda concatemers (New England Biolabs, Ipswich, MA) were used as size standard. Gels were stained with ethidium bromide and digitalized using the Kodak Digital Science system (Kodak, Milan, Italy).

### PCR-based fingerprinting using random amplified polymorphic DNA (RAPD)

Genotyping of AIEC isolates was performed using RAPD PCR. Bacterial DNA was purified using mericon DNA Bacteria Kit (Qiagen, Italy) according to the manufacturer’s instructions. RAPD reactions were performed using two arbitrary primers; primer 3 (5′-d[GTAGACCCGT]-3′) and primer 4 (5′-d[AAGAGCCCGT]-3′) [30]. Each lyophilized primer was rehydrated in sterile distilled water prior to use. Amplification was performed in a 25-μL reaction mixture containing 1.5 mM MgCl_2_ (Bioline, London, UK), 0.2 mM dNTPs (Promega, Madison, WI, USA), 0.5 μM of each primer, 1U BioTaq™ of DNA polymerase in 1X PCR buffer (Bioline, London, UK) and 3 μL of template dsDNA (40 ng). PCR was performed as follows: 1 cycle at 95°C for 5 min, followed by 45 cycles at 95°C for 1 min, 36°C for 1 min, and 72°C for 2 min (Mastercycler pro, Eppendorf Germany). Ten microliters of all reactions were electrophoresed in 2.0% agarose gels, stained with ethidium bromide and photographed by use a ultraviolet transilluminator and a digital capture system (UVP Inc.) In order to guarantee reproducibility of RAPD profiles, each PCR reaction was performed in triplicate.

### PFGE and RAPD profiles analysis

The DNA banding patterns obtained by PFGE and RAPD assays were analysed with TotalLab TL120 Trace version 2006 (Nonlinear Dynamics) with a position tolerance set at 1,5%. The Dice coefficient of similarity was calculated, and the unweighted pair group method with arithmetic averages (UPGMA) was used for cluster analysis XLstat 7.5 (Addinsoft, USA). The similarity percentage cut-off to distinguish between clonally distinct groups was set at 95%.

### Adhesion phenotypes assay

The pattern of adhesiveness of our AIEC-like strains was evaluated using HEp-2 and Caco-2 cell monolayers, localized adherence (LA), diffuse adherence (DA) and enteroadherent-aggregative adherence (AA) were determined for all the AIEC genotypes by the method described by Scaletsky et al. [31]. Monolayers were infected with bacterial suspensions (10^6^ bacteria/ml; MOI = 10) and incubated at 37°C for 3 h. Infected monolayers were examined at light microscope after a 3-h incubation period. When needed, bacterial cultures were harvested in the exponential phase and suspended in culture medium with and without 0.5% (wt/vol) D-mannose (Sigma-Aldrich, Italy).

### Phylotyping

The phylogenetic group of all *E. coli* AIEC genotypes was assayed by a multiplex PCR protocol [32]. The oligonucleotide primers were designed to amplify the *E. coli* associated genes *chuA, yjaA* and *TspE4C2*. Phylogenetic clustering was made on the basis of the results of amplification. Namely group A was *chuA–, yjaA +/-, TspE4C2*–*,* group B1 *chuA–, yjaA+/–, TspE4C2+*, group B2 *chuA+, yjaA+, TspE4C2+/*–, group D *chuA+, yjaA–, TspE4C2+/–*. Amplifications were carried out in 25 μl reaction mixtures composed of 1X PCR reaction buffer (Biolabs Inc.), 50 ng/μl of template DNA, 0.2 mM of dNTPs (Biolabs Inc.), 0.5 μM of primers (Sigma), 1.25 U Taq DNA polymerase (Biolabs Inc.). Amplification was carried out for 30 cycles each consisting of a denaturing step of 5 min at 95°C, followed by an annealing step of 30 s at 56°C and an extension step of 5 min at 72°C. The amplification products were separated by electrophoresis in 2.0% agarose gels, in 45 mM Tris-borate, 1 mM EDTA buffer (pH = 8.0) containing ethidium bromide at 0.5 μg/ml at a constant voltage of 5 V/cm. Gels were photographed under transillumination using a digital camera (UVP Inc.) Amplification was performed in triplicate. The size of amplicons was determined by comparison with a 100-bp DNA ladder (Biolabs inc.).

### Virulence genotyping by PCR

All the AIEC genotypes were assayed for selected virulence and fimbrial adhesin genes known to be associated with virulent *E. coli* strains by multiplex PCR using appropriate primers [see Table 1]. Whole DNA bacterial extracts were prepared using Qiagen DNA extraction kit (Qiagen, Italy). *E. coli* strains known to encode the assayed virulence traits were included as controls. Amplifications were carried out for 30 cycles in 25 μl in 1X reaction buffer (Euroclone). Each reaction mixture contained 50 ng/μl of each of template DNA, 1.5 mM MgCl_2_, 0.2 mM of dNTPs (Biolabs Inc.), 0.5 μM of each primer pairs, 1.25 U of Taq DNA polymerase (Euroclone). For some virulence genes, namely *gafD, cvaC, focG, traT* and *papG* allele II; *sfa/focDE*, *iutA, papG* allele III) the multiplex PCR amplification was carried out in a total volume of 50 μl, using 5 μl of whole bacterial DNA extracts as templates. The amplification products were separated by electrophoresis in 2.0% agarose gels, as described above and stained with ethidium bromide. Gels were photographed using a digital camera (UVP Inc.) Amplification was performed in triplicate. The size of amplicons was determined by comparison with a 100-bp DNA ladder (Biolabs Inc.).

**Table 1:** **Primers used for PCR analysis of known virulence genes of extra-intestinal pathogenic** ***E. coli***

Virulence traits	Genes	Primer sequence (5′à 3′)	Expected amplicon size (bp)	References
**P fimbriae**	*papA*	ATGGCAGTGGTGTCTTTTGGTG	720	[33]
		CGTCCCACCATACGTGCTCTTC		
	*papEF*	GCAACAGCAACGCTGGTTGCATCAT	336	[33]
		AGAGAGAGCCACTCTTATACGGACA		
**P pili-associated G-adhesins**	*papC*	GACGGCTGTACTGCAGGGTGTGGCG	328	[33]
		ATATCCTTTCTGCAGGGATGCAATA		
**Pyelonephritis-associated pili**	papG allele II e III	CTGTAATTACGGAAGTGATTTCTG	1070	[33]
		ACTATCCGGCTCCGGATAAACCAT		
	papG allele II	GGGATGAGCGGGCCTTTGAT	190	[33]
		CGGGCCCCCAAGTAACTCG		
	papG allele III	GGCCTGCAATGGATTTACCTGG	258	[33]
		CCACCAAATGACCATGCCAGAC		
**S and F1C fimbriae**	*sfa//focDE*	CTCCGGAGAACTGGGTGCATCTTAC	410	[33]
		CGGAGGAGTAATTACAAACCTGGCA		
**F1C fimbriae**	*focG*	CAGCACAGGCAGTGGATACGA	360	[33]
		GAATGTCGCCTGCCCATTGCT		
**Dr-binding adhesin**	*afa/draBC*	GGCAGAGGGCCGGCAACAGGC	559	[33]
		CCCGTAACGCGCCAGCATCTC		
**G fimbriae**	*gafD*	TGTTGGACCGTCTCAGGGCTC	952	[33]
		CTCCCGGAACTCGCTGTTACT		
**Nonfimbrial adhesin type 1**	*nfaE*	GCTTACTGATTCTGGGATGGA	559	[33]
		CGGTGGCCGAGTCATATGCCA		
**Mannose-specific adhesion gene. Type1 fimbriae.**	*fimH*	TGCAGAACGGATAAGCCGTGG	508	[33]
		GCAGTCACCTGCCCTCCGGTA		
**Haemolysin A**	*hlyA*	AACAAGGATAAGCACTGTTCTGGCT	1177	[33]
		ACCATATAAGCGGTCATTCCCGTCA		
**Cytotoxic necrotizing factor 1**	*cnf1*	GTGATAATATATCACATTATTC	498	[33]
		GAATTCGTCTCGTTGAGCTTCACTG		
**Yersiniabactin siderophore receptor**	*fyuA*	TGATTAACCCCGCGACGGGAA	880	[33]
		CGCAGTAGGCACGATGTTGTA		
**Aerobactin siderophore receptor**	*iutA*	GGCTGGACATCATGGGAACTGG	300	[33]
		CGTCGGGAACGGGTAGAATCG		
**Capsule synthesis primers**	*kpsMT II*	GCGCATTTGCTGATACTGTTG	272	[33]
		CATCCAGACGATAAGCATGAGCA		
	*kpsMT III*	TCCTCTTGCTACTATTCCCCCT	392	[33]
		AGGCGTATCCATCCCTCCTAAC		
	*kpsMT1 (K1)*	TAGCAAACGTTCTATTGGTGC	153	[33]
		CATCCAGACGATAAGCATGAGCA		
	*kpsMT 5 (K5)*	CAGTATCAGCAATCGTTCTGTA	159	[33]
		CATCCAGACGATAAGCATGAGCA		
**Invasion of brain endothelium gene (associate with neonatal meningitis)**	*IbeA*	AGGCAGGTGTGCGCCGCGTAC	170	[33]
		TGGTGCTCCGGCAAACCATGC		
**Encodes colicin V**	*cvaC*	CACACACAAACGGGAGCTGTT	680	[33]
		CTTCCCGCAGCATAGTTCCAT		
**Serum resistance gene**	*traT*	GGTGTGGTGCGATGAGCACAG	290	[33]
		CACGGTTCAGCCATCCCTGAG		
**Pathogenicity islands described in a virulent uropathogen.**	*PAI*	GGACATCCTGTTACAGCGCGCA	930	[33]
		TCGCCACCAATCACAGCCGAAC		
**Transcriptional Activator gene of aggregative adhesion fimbriae**	*aggR*	GTATACACAAAAGAAGGAAGC	254	[34]
		ACAGAATCGTCAGCATCAGC		
**CVD432 gene probe sequence of the plasmid of aggregative adhesion**	*pAA (CVD432)*	CTGGCCAAAGACTGTATCAT	630	[34]
		CAATGTATAGAAATCCGCTGTT		

### Statistical methods

The differences in the distribution of phylogroups of AIEC strains, between the two populations of subjects studied, were calculated using the *χ*2 test with Yates’ continuity correction. For Dice similarity index a bilateral Mann-Whitney *U*-test was utilized to compare patients and controls. In both cases, a *P* value ≤0.05 was considered statistically significant.

## Results

### High abundance of adherent *E. coli*isolates from CD patients

Six-hundred sixteen independent *E. coli* isolates were collected from ileal mucosa biopsies from four pediatric CD patients and from four non-IBD controls. Isolates were assayed for their ability to adhere to HEp-2 and Caco-2 cell monolayers (as described in Materials and Methods) and a total of 226 adherent *E. coli* isolates were detected. The relative abundance of adhesive isolates was significantly higher among isolates from CD patients than non-IBD controls: 63% (161/255) vs 23% (65/273) (*χ*2 = 81.69 *P* < 0.001), respectively. We could not determine the adhesive ability of 88 isolates (53 *E. coli* isolates from CD patients, and of 35 from non-IBD controls) since they were highly cytotoxic when challenged with tissue culture (detachment and lysis of cell monolayers was observed 3 h post infection) (data not shown). Analysis of these 88 isolates revealed that all produced higher levels of hemolysin, and the release of hemolysin may likely account for the high cytotoxic activity of these isolates [34]. These strains were not used for further analysis.

### Invasive *E. coli*isolates were able to replicate within macrophages

Invasiveness of the 226 adherent *E. coli* isolates was tested by the gentamicin protection assay using HEp-2 cell monolayers [13]. At the end of the gentamicin treatment, infected cell monolayers were extensively washed, lysed, and the number of intracellular bacteria determined for each isolate (see Materials and methods for details). Isolates were considered invasive when the CFUs of each invasive bacteria was superior or equal to 0.1% of the number of bacteria used to infect cell monolayers (MOI = 10). Reference *E. coli* K-12 strain DH5α and reference AIEC strain LF82 were used an non-invasive and invasive controls, respectively. Under our experimental conditions DH5α presented an invasive score of 0.0007% ±0.0005 while LF82 a score of 0.95% ±0.3 in respect of the initial bacterial inoculum (Table 2). Of the 226 adhesive isolates, 36 were also invasive (invasive score ranged from 0.40% ±0.11% to 0.88% ±0.27%) and of these 31 isolates were from three of the four CD patients and 5 from two of the four non-IBD controls. The relative abundance (% AIEC isolates/total *E. coli* isolates) was significantly higher among isolates from CD patients (31/308; 10.06%) than in non-IBD controls (5/308; 1.62%) (*χ*2 = 18.43 p < 0.0001). Furthermore, the relative abundance (% AIEC isolates/total *E. coli* isolates for each patient) was 14/77 (18,18%), 9/77 (11,68%), 8/77 (10,38%), 0/77 (0%) in CD1,CD2,CD3 and CD4 respectively (mean ± SD; 10,06 ± 7,52) and 4/77 (5,19%), 1/77 (1,29%), 0/77 (0%), 0/77 (0%) in controls 1,2,3 and 4 respectively (mean ± SD; 1,62 ± 2,45).

**Table 2:** **HEp-2 invasiveness of the 36 AIEC isolated from patients with CD (CD1-4) and from non-IBD controls (Control 1-4)**

Sample origin	N of invasive strains	^a^(%) Average invasion level
CD1	14	0.50 ± 0.15
CD2	9	0.40 ± 0.11
CD3	8	0.88 ± 0.27
CD4	0	–
Control 1	4	0.67 ± 0.10
Control 2	1	0.52 ± 0.15
Control 3	0	–
Control 4	0	–
Reference strains	*E. coli* LF82	0.95 ± 0.30
	*E. coli* DH5α	0.007 ± 0.005

^a^Values represent the mean percentages ± standard deviations of the means of internalised bacteria and are expressed as the ratio of intracellular CFU/number of bacteria used to infect cell monolayers X 100. Data represent mean and standard errors of three independent experiments, in duplicate.

--, not determined.

To assess whether the 36 adhesive-invasive isolates were putative AIEC-like isolates, they were further assayed for the ability to survive and multiply within macrophages. The number of bacteria surviving the gentamicin treatment was evaluated at 1 and 24 h post-infection. As expected, when challenged with J774 macrophages, the non-invasive K-12 DH5α strain was rapidly eliminated, while LF82 retained its ability to survive and multiply within macrophages. Remarkably, all the 36 adherent-invasive *E. coli* isolates, were able to survive and multiply within J774 macrophages (Table 3). This finding clearly indicated that they may well be considered AIEC-like isolates [17].

**Table 3:** **Thirty-six adherent/invasive** ***E. coli*** **isolates were able to persist and to replicate within J774 macrophages**

Sample origin	N of isolates	^a^Percentage of survival/replication in J744 macrophages
CD1	14	403 ± 102
CD2	9	638 ± 113
CD3	8	417 ± 133
Control 1	4	376 ± 67
Control 2	1	340 ± 110
Controls strains	*E. coli* LF82	580 ± 120
	*E. coli* DH5α	7.3 ± 4.0

^a^Percentage of intracellular bacteria at 24 hours post infection relative to the number of bacteria after 1 h of gentamicin treatment, defined as 100%. CD1-3, CD patients; Control 1-2; non-IBD patients. Data represent mean and standard errors of three independent experiments made in duplicate.

### Genotypic variants among AIEC isolates

**Figure 1:**
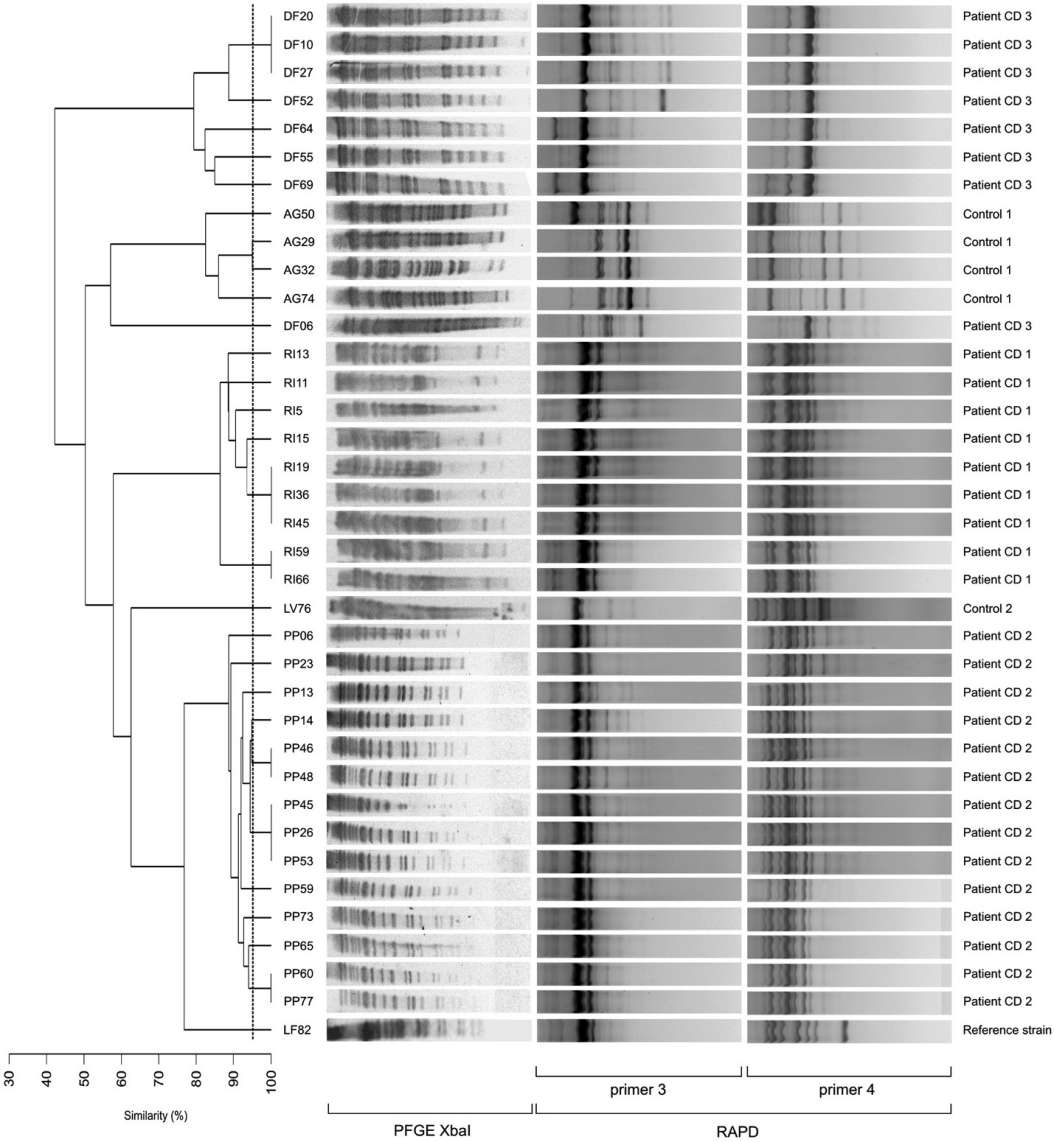
**Genetic variability among the 36 AIEC isolates from CD patients CD1, CD2 and CD3, and from non-IBD controls Control-1 and Control-2.** Consensus UPGMA dendrogram, generated on Dice coefficients of *Xba*I PFGE and RAPD-PCR combined profiles (similarity cut-off level 95%) identified 27 genetic variants (22 from CD patients and 5 for controls). Genotypes of AIEC strains isolated from the same patient tended to cluster.

**Figure 2:**
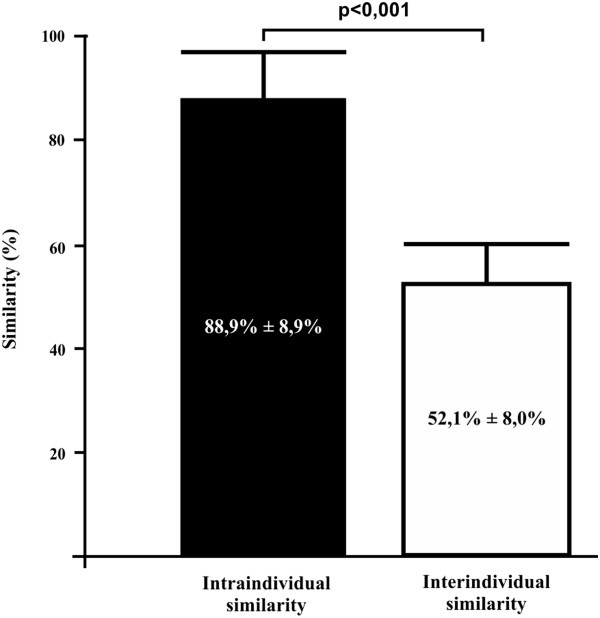
**Similarity analysis between AIEC genotypes isolated from CD patients and non-IBD controls.** Percentage values were based on Dice similarity index. A *P* value ≤0.05 was considered statistically significant.

PFGE and RAPD fingerprinting analysis was conducted in order to genotypically characterize the 36 AIEC-like isolates and a dendrogram was constructed (Figure 1). Using this approach, we detected 27 different genotypes among the 36 AIEC-like isolates (22 were from CD patients and 5 from non-IBD controls). Genotypes belonging to the same patient tended to cluster. Six to 10 genetic variants (mean ± SD; 7.3% ±2.3%) were detected among the 22 AIEC-like strains isolated from CD patients, while 1 to 4 (mean ± SD: 2.5 ± 2.1) were obtained from AIEC-like strains isolated from non-IBD controls. Although the differences in genotypic richness are not statistically significant, because of the small number of patients enrolled in this study, these results may suggest a higher diversity among AIEC strains isolated from CD patients compared to those isolated from non-IBD controls. The similarity level among the 27 genotypes, computed by calculating the Dice index, showed a statistically significant difference (*P* <0.0001) between intra-individual similarity (88.9% ±8.9%) and inter-individual similarity (52.1% ±8.0%) (Figure 2). Due to the small number of patients enrolled in this study these differences cannot be considered significant although they are in agreement with previous findings suggesting a higher diversity among AIEC strains isolated from CD patients. Further study is surely needed to clarify this point.

### All the AIEC-like strains show an aggregative (AA) pattern of adherence

**Figure 3:**
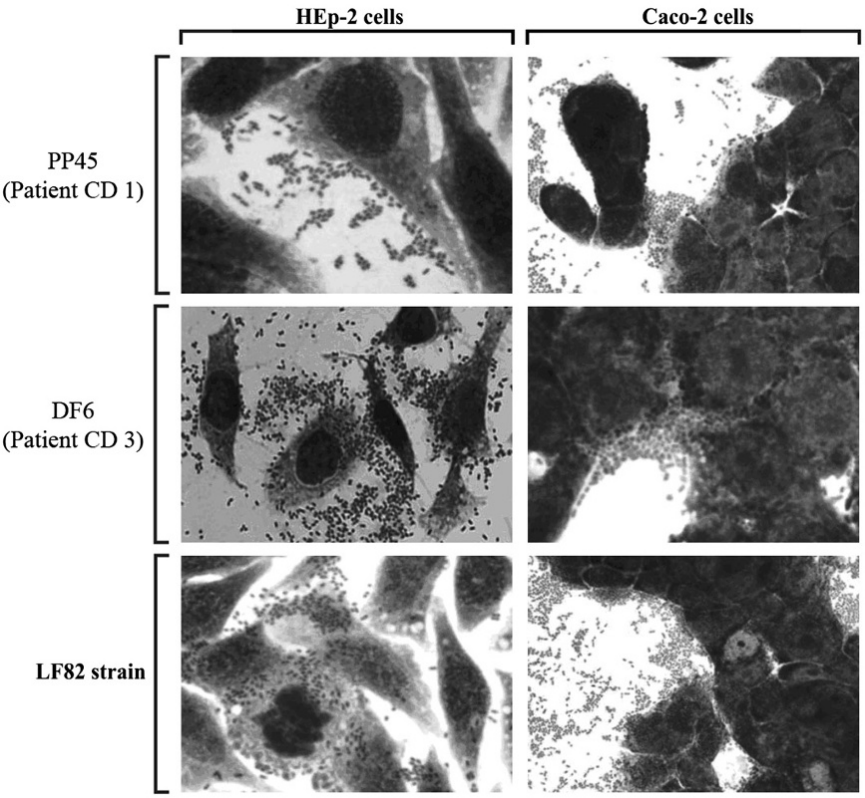
**Adhesion phenotypes assay.** Phase contrast fields showing Hep-2 and Caco-2 cell monolayers infected with two representative AIEC strains isolated in this work (PP45 and DF6) and with reference AIEC LF82 strain. All 27 AIEC strains showed an aggregative (AA) patterns of adherence. Magnification, 1000 X.

The pattern of adherence of the 27 AIEC-like strains was determined using HEp-2 as well as Caco-2 cell monolayers. Remarkably, as AIEC strain LF82, all the 27 AIEC-like strains presented an aggregative pattern of adherence (AA) (Figure 3) that was inhibited by co-culturing bacteria and both type of epithelial cells with D-mannose (data not shown). These data are in agreement with previous work indicating that expression of type 1 pili is necessary for AIEC adhesiveness [19, 35].

### Phylogenetic grouping and virulence genetic markers of the AIEC-like isolates

The phylogenetic background of the 27 AIEC genotypes was examined by PCR grouping analysis, as previously described [32]. The 22 AIEC-like genotypes isolated from CD patients were phylogenetically classified as follows: 40.9% (9/22) belonged to group A, 31.8% (7/22) belonged to group D, while only 18.2% (4/22) and 9.1% (2/22) belonged to groups B1 and B2, respectively. Of the five AIEC strains isolated from the non-IBD controls, 80% (4/5) belonged to group A and 20% (1/5) to group B2. Although it has previously reported that the B2 and D phylogroups were the most prevalent among AIEC isolates [16, 36], our result did not show significant differences in phylogroup predominance and in phylogroups distribution in IBD compared to controls. With the exception of *fimH* that was detected in all the 27 AIEC genotypes, significant differences in the presence of virulence gene were found between the AIEC genotypes isolated from CD patients and the non-IBD controls (Table 4). Furthermore we found that virulence gene profiles were more similar in genotypes coming from the same patient being the intra-individual similarity (89,3% ±12,2%) statistically different from. inter-individual one (53,0% ±28,1%) (p < 0,0001).

**Table 4:** **Prevalence of virulence genes, identified for each strain by PCR, in the 22 AIEC strains in CD and in the 5 non-IBD pediatric patients**

Virulence Factors		% of virulence genes in AIEC isolates from CD patients (n = 22)	% of virulence genes in non-IBD controls (n = 5)	^a^P value
Adhesins	*pap*A	0,00%	0,00%	–
	*pap*C	0,00%	0,00%	–
	*pap*EF	0,00%	0,00%	–
	*pap*G	0,00%	0,00%	–
	*pap*G allele II	13.63%	0,00%	NS
	*pap*G allele III	0,00%	0,00%	–
	*pap*G allele II e III	0,00%	0,00%	–
	*sfa/foc*DE	0,00%	0,00%	–
	*foc*G	0,00%	0,00%	–
	*fim*H	100.00%	100.00%	NS
	*Afa/Dra*BC*,*	0,00%	0,00%	–
	*nfa*E	0,00%	0,00%	–
	*gaf*D	0,00%	0,00%	–
Capsule	*K*1	72.72%	0,00%	0.013
	*K*5	4.54%	0,00%	NS
	*kps*MT II	72.72%	0,00%	0.013
	*kps*MT III	0,00%	0,00%	–
	*rfc*	0,00%	0,00%	–
Siderophores	*fyu*A	72.72%	0,00%	0.013
	*iut*A	0,00%	0,00%	–
Toxins	*cnf*1	0,00%	0,00%	–
	*cva*C	0,00%	0,00%	–
	*hly*A	0,00%	0,00%	–
Invasin	ibeA	81.81%	0,00%	0.001
Pathogenic island	*PAI*	0.00%	0,00%	–
Resistance to serum	*tra*T	54.54%	0,00%	NS
Transcriptional Activator gene of aggregative adhesion fimbriae	*aggR*	4,54%	0,00%	NS
CVD432 gene probe sequence of the plasmid of aggregative adhesion	*pAA (CVD432)*	0,00%	0,00%	–

^a^NS, not significative.

--, not determined.

## Discussion

In this work, a large number of *E. coli* isolates obtained from ileal mucosa biopsies from four pediatric CD patients and from four non-IBD controls (77 isolates per biopsy, for a total of 616 independent isolates) were characterized. Isolates were analyzed to evaluate the presence and the abundance of AIEC strains. The ability to adhere, to invade HEp-2 cell monolayers and to survive and multiply within macrophages (typical AIEC characteristics) were detected for 36 *E. coli* isolates (AIEC-like strains). We noticed a significant higher relative abundance of AIEC-like isolates among biopsies of CD patients (31 isolates vs five isolates from non-IBD controls), supporting previous findings indicating a close association of AIEC strains with CD patients intestinal mucosa [16, 37]. Combined RAPD and PFGE fingerprints analysis, conducted on the 36 AIEC-like isolates, evidenced 27 different genotypes, each of which appeared to be unique for each patient. Indeed, patient CD1 harboured up to 10 distinct AIEC genotypes while patients CD2 and CD3 both harboured 6 distinct genotypes, while four and one distinct genotypes were found for AIEC strains isolated from Control 1 and 2, respectively. Furthermore, the dendogram, shown in Figure 1, also indicates that AIEC strains isolated from the same patients are closely related. Alike reference AIEC LF82 strain, all the 27 AIEC genotypes presented an AA pattern of adherence on HEp-2 cells. This result, led us to speculate that this pattern could favor the intestinal colonization and persistence within the inflamed intestinal mucosa of CD, at least in pediatric CD patients [16, 34].

In contrast with studies regarding adult CD patients which reported phylogroup B2 as the predominant one [16], phylogroup B2 was not predominant among our AIEC-like strains. Probably due to the small number of patients examined, we did not detect any significant phylogroup predominance or differences in phylogroup distribution. Nevertheless, we have previously reported [38] a very low proportion of B2 strains in AIEC strains of pediatric origin and isolated in Italy, suggesting that age and country of provenience may well be the cause of this discrepancy. With the exception of *fimH*, PCR-based identification of virulence genes known to be present in extra-intestinal pathogenic *E. coli*, indicated that only AIEC-like strains isolated from CD patients were positive for the presence of genes involved in the synthesis of capsular material (*K*1, *K*5 and *kps*MT II), in the production of siderophores (*fyuA*), invasiveness (*ibeA*) and resistance to serum (*traT*). No CD-associated AIEC isolates was found positive for *cnf1*. Again, these results are in contrast with those reported by Martinez-Medina et al., [16] who found that the virulence gene content of mucosa-associated AIEC strains isolated from adult CD patients was similar to healthy controls. The finding that we detected virulence genes only in AIEC-like strains isolated from CD patients may be due to the small number of patients enrolled in this study or that by the use of pediatric patients.

## Conclusions

In conclusion, the AIEC-like abundance in CD patients and the presence of virulence genes only in CD patients further support the hypothesis that this pathovar may play an etiologic role in the insurgence and/or in the persistence in CD. These results are further supported by the recent findings indicating that colonization of the intestinal mucosa by AIEC strains, may initiate chronic inflammation of susceptible hosts by altering the gut microbiota [23]. Moreover, it has been recently reported a higher incidence of IBD in patients feed with fat-rich Western diet. It has been suggested that a fat-rich diet may induce changes in the composition of intestinal microbiota that, in turn, may influence the colonization and persistence of AIEC strains in genetically susceptible hosts [39]. These findings open up new scenarios addressed at the development of new strategies for the prevention and control of IBD not only based at eliminating AIEC strains from the inflamed intestinal mucosa but also throughout calibrated nutritional interventions.
